# The Role of Small-Bowel Capsule Endoscopy in the Diagnostic Algorithm of Complicated Perianal Disease

**DOI:** 10.3390/diagnostics14161733

**Published:** 2024-08-09

**Authors:** Irit Avni-Biron, Ervin Toth, Jacob E. Ollech, Artur Nemeth, Gabriele Wurm Johansson, Hagai Schweinstein, Reuma Yehuda Margalit, Uri Kopylov, Iris Dotan, Henit Yanai

**Affiliations:** 1 Division of Gastroenterology, Rabin Medical Center, Petah Tikva 4941492, Israel; 2 Sackler Faculty of Medicine, Tel Aviv University, Tel Aviv 6997801, Israel; 3 Department of Gastroenterology, Skåne University Hospital, Lund University, 21428 Malmö, Sweden; 4 Department of Clinical Sciences, Lund University, 20502 Lund, Sweden; 5 Institute of Gastroenterology, Sheba Medical Center, Tel Hashomer, Ramat Gan 5266202, Israel

**Keywords:** capsule endoscopy, perianal Crohn’s disease, diagnosis, algorithm, fecal calprotectin

## Abstract

Introduction: Complicated perianal disease (cPD) may be the sole presentation of Crohn’s disease (CD). The role of small-bowel capsule endoscopy (SBCE) in the diagnostic algorithm of cPD is unclear. We aimed to evaluate the role of SBCE as a diagnostic tool, in patients with cPD, after a negative standard workup for CD. Methods: A multicenter, retrospective, cross-sectional study, in patients with cPD, and negative standard workup for CD (ileocolonoscopy and cross-sectional imaging), who underwent SBCE for suspected CD. Demographics, biomarkers, and the Lewis Score (LS) were recorded and analyzed. An LS ≥ 135 was considered a positive SBCE for diagnosing CD. Results: Ninety-one patients were included: 65 (71.4%) males; median age: 37 (29–51) years; cPD duration: 25.1 (12.5–66.1) months. Positive SBCE: 24/91 (26.4%) patients. Fecal calprotectin (FC) positively correlated with LS (*r* = 0.81; *p* < 0.001). FC levels of 100 µg/g and 50 µg/g had a sensitivity of only 40% and 55% to rule out small-bowel CD, with a negative predictive value (NPV) of only 76% and 80%, respectively. Conclusions: SBCE contributed to CD diagnosis in a quarter of patients with cPD after a negative standard workup. FC levels correlated with the degree of inflammation defined by the LS. However, the NPV of FC was low, suggesting that SBCE should be considered for patients with cPD even after a negative standard workup.

## 1. Introduction

Complex perianal disease (cPD) may result from cryptoglandular disease or Crohn’s disease (CD). In 4% of patients with CD, cPD may precede or be the sole manifestation of CD [1,2]. The diagnosis of CD in patients with cPD is essential, as it will impact management strategies; a biologic medication may be added to control inflammation [3,4,5,6], mesenchymal stem cells (MSCs) may be injected along the fistula tract, and a monitoring strategy will be implemented to assess disease progression [7,8,9,10].

**Figure 1:**
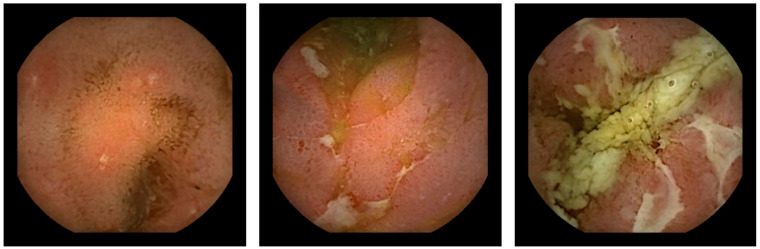
Capsule endoscopic findings of Crohn’s lesions.

The diagnostic algorithm for suspected CD includes ileocolonoscopy as the first diagnostic tool. When ileocolonoscopy is reported as normal, but CD is still suspected, further evaluation is executed with dedicated cross-sectional imaging, followed by small-bowel capsule endoscopy (SBCE) [11]. SBCE was shown to be a sensitive tool for the detection of mucosal lesions (Figure 1), revealing proximal disease, and leading to a change in disease phenotype in a substantial proportion of CD patients [12,13,14].

Additionally, fecal calprotectin (FC) has been increasingly used in the investigational algorithm of bowel inflammation. The optimal FC cutoff depends on the screened population, type of IBD, disease location, selected outcome, and the diagnostic modality used [15,16,17]. The value of FC as a selection tool for further investigation by SBCE in patients with cPD after a negative standard workup has not been investigated.

Since SBCE is a highly sensitive tool for the detection of small-bowel mucosal lesions [18], we aimed to evaluate its role in the diagnostic algorithm of small-bowel CD for patients with cPD after a negative standard workup for CD.

## 2. Materials and Methods

### 2.1. Setting and Participants

A multicenter, retrospective, cross-sectional study was conducted in three large tertiary centers serving as IBD referral centers and providing capsule endoscopy services: a Malmö gastroenterology center in Sweden, and Rabin and Sheba medical centers in Israel. In each center, a thorough review of the SBCE database was performed to identify relevant cases. All centers used the PillCam™ SB3 capsule platform (PillCam, Medtronic, Dublin, Ireland). Images were reviewed using the RAPID™ Reader Software v8 or 9. All capsule SBCE examinations were evaluated by expert readers (>1000 cases), according to ESGE standards [19], with one reader per examination.

Patients with cPD, who were referred by their treating physician to an SBCE study to evaluate suspected CD after a negative ileocolonoscopy, and negative cross-sectional imaging (CT enterography or MR enterography) were included. Patients with perianal fissures as a sole presentation of perianal disease were excluded. We recorded laboratory data up to 6 months before the SBCE study date. Post-procedure therapy, including administration of anti-TNF therapy and mesenchymal stem cell (MSC) injection administration, were recorded.

### 2.2. Outcomes and Definitions

The primary outcome was the proportion of patients with small-bowel inflammation detected by SBCE leading to a diagnosis of CD.

The secondary outcome was to investigate the value of FC as a selection tool for further investigation with SBCE in patients with cPD, and to identify a cutoff value for FC that could obviate the need for further evaluation with SBCE.

cPD was defined as one or more of the following: high inter-sphincteric, high trans-sphincteric, extra-sphincteric, supra-sphincteric fistula tracts, or those associated collections/abscesses, as per criteria established by the American Gastroenterological Association [20].

Mucosal inflammation was quantified using the Lewis Score (LS) [21]. A score < 135 indicates normal or clinically insignificant mucosal inflammatory changes, an LS of 135–790 indicates mild inflammation, and a score of >790 indicates moderate-to-severe inflammation [21]. Thus, positive SBCE was defined as an LS ≥ 135. In addition to the traditional LS (derived from the score achieved by the most involved tertile), we also collected data of the individual tertile scores. The proximal small bowel was defined as the first or second tertile in accordance with small-bowel transit times.

### 2.3. Statistics

Descriptive statistics were presented as medians with an interquartile range (IQR) for continuous variables and percentages for categorical variables. A two-tailed *p* < 0.05 was considered statistically significant. The relationship between variables was assessed by calculating the Pearson correlation test. To estimate the discriminatory effects of biomarkers to assess a positive SBCE, we used the receiver operating characteristics (ROC) analysis. The test metrics sensitivity, specificity, positive predictive value, and negative predictive value (NPV) were calculated. Regression analysis was used to assess risk between biomarkers and positive SBCE. Analysis was performed using IBM SPSS statistics, version 28.0, IBM Corp., Armonk, NY, USA, 2021.

## 3. Results

Ninety-one patients were included: 65 males (71.4%); median age: 37 (29–51) years. cPD duration was 25.1 (12.5–66.1) months. Per inclusion criteria, all participants had complicated perianal fistulizing disease, with a negative ileocolonoscopy and a negative cross-sectional small-bowel analysis and were referred for SBCE for the investigation of suspected small-bowel CD; biomarkers were obtained within 6 months prior to the SBCE study (145 (112–213) days for FC). Biomarkers were mostly within the normal range (Table 1 for the complete panel of biomarkers). Specifically, median FC was 29 µg/g (IQR: 10–105, *n* = 71); FC was above 50 µg/g in 29/71 (40.8%) patients, and above 100 µg/g in 18/71 (25.4%) patients.

Positive SBCE was identified in 24/91 (26.4%) patients (Table 2 for details).

The median LS was 675 (222–1518); moderate–severe LS was demonstrated in 10/24 (41.6) patients. Post SBCE, follow-up was available for a median of 56 (22.36–88.46) months. CD diagnosis was accepted by the treating physician based on SBCE findings in 23/24 patients with a positive SBCE. In one patient, further evaluation was planned with repeated SBCE.

Post-CD diagnosis, anti-TNF therapy was recommended to 14/24 patients (9 with LS > 790 and 5 with LS < 790), and MSC therapy was administered to three (one with LS > 790, and two with LS < 790).

Hemoglobin and CRP levels were weakly correlated with LS (*r* = −0.14; *p* = 0.53 and *r* = 0.25; *p* = 0.30). Conversely, FC was strongly correlated with LS (*r* = 0.82; *p* < 0.001). Median FC was 10 µg/g (10–113) in the sub-group of patients with mild inflammation (LS < 790, *n* = 9) and 150 (40–579) in the sub-group of patients with moderate–severe inflammation (LS > 790, *n* = 10), *p* = 0.159. In regression analysis, only FC was associated with a positive SBCE test result (regression coefficient 3.626, 95% CI: 2.355–4.897, *p* < 0.001).

Discriminating positive vs. negative SBCE by FC levels revealed an area under the curve (AUC) of 0.61 (95% CI 0.444 to 0.769, *p* = 0.163). FC cutoff of 300 µg/g had a specificity of 90.2% for positive SBCE; however, an FC cutoff of 100 µg/g had a sensitivity of 40% for ruling out a positive SBCE, and an FC cutoff of 50 µg/g had a sensitivity of 55%. The negative predictive value for FC values of 100 µg/g and 50 µg/g was 78.9% and 80.1%, respectively (Table 3). Finally, an ROC analysis to investigate the discriminatory effect of FC level for positive SBCE based on an LS ≥ 350 vs. values below 350 revealed an AUC of 0.52 (95% CI 0.180–0.879, *p* = 0.873).

## 4. Discussion

In this cohort of 91 patients with cPD, negative ileocolonoscopy, negative cross-sectional imaging, and mostly normal biomarker levels, positive SBCE was noted in 26.4% (24/91). This contributed to establishing a diagnosis of CD in 23 patients (96%), affecting consequent management.

CD was diagnosed by SBCE in 24% of patients presenting with perianal disease after a negative standard workup, in a small study of 26 patients, conducted more than a decade ago [22]. The same proportion (26%) was once again reported by another, recent, retrospective study [23]. In 12/45 of patients with cPD, SBCE was positive, compared to 3% (3/90) of patients referred for SBCE for other indications [23]. The current study supports this proportion of SBCE positivity in this patient population in a larger group of patients.

SBCE contributed to the identification of proximal small-bowel inflammatory involvement in 16/24 (67%) of patients. The superiority of SBCE in identifying superficial and proximal mucosal inflammation was well established in previous reports [13,24,25,26]. It was also previously recognized that a discrepancy may exist in the detection of small-bowel lesions between the various modalities. In a study that compared the quantification of distal SB inflammation by VCE- and MRI-related activity indices in quiescent CD patients, the area under the curve (AUC) with both MRI scores was only moderate for the prediction of any mucosal inflammation (LS ≥ 135) [27]. The diagnostic accuracy of MRE can also be affected by several technical aspects: imaging artifact due to motion, bowel peristalsis and precision in intake, and the timing of oral contrast [24]. While ileocolonoscopy is an essential tool for CD diagnosis, small-bowel CD may still be recognized in patients with negative ileocolonoscopy. In a study of 67 pediatric CD patients with normal results from ileoscopy, 36 (53.7%) had active, small-bowel Crohn’s disease identified by isolated histologic, intramural, or proximal inflammation [28,29].

The identification of luminal inflammation by SBCE had important clinical implications, as CD-related therapy was initiated in most patients (71%, 17/24). SBCE contribution to the change in management was most prominent among patients with moderate–severe luminal inflammation (100%). Among patients with mild luminal inflammation detected by SBCE (LS < 790), CD-related therapy was initiated in 50% of patients (7/14). The natural history of mild luminal small-bowel CD diagnosed by SBCE only is not well established and investigated, and further inquiry is needed for better understanding this phenotype of patients. Indeed, a lower acceptance of treatment modification was demonstrated in cases of LS < 790. In support, among the 12/45 patients with cPD and luminal inflammation detected by CE in the study by Mccurdy et al., 58% (7/12) were recommended immunosuppressive therapy [23].

In patients with suspected CD after a negative ileocolonoscopy, the diagnostic utility of FC as a selection tool for SBCE remains controversial. While one study demonstrated a negative predictive value of 100% when FC was <100 mg/kg [30], in another, a negative FC could not rule out active small-bowel CD in 21% of patients [31]. We aimed to determine the diagnostic utility of FC as a selection tool for SBCE in patients with cPD, by comparing its values with the inflammatory activity determined by LS. FC demonstrated a strong correlation with positive SBCE. However, sensitivity and NPV were low (either 100 or 50 µg/g), and both cutoffs could not obviate the need for SBCE, even when testing it for a higher LS of >350 [32]. A similar conclusion was drawn in a retrospective study of 161 patients with suspected CD. While a high FC value implied a higher probability of positive SBCE, FC alone could not be used as a selection tool for SBCE in patients with suspected small-bowel CD in a specialist setting [33]. In the current study, like other cohorts, CRP and hemoglobin were weakly associated with positive SBCE [18,33,34].

Our study has several strengths; it is multicenter, focusing on a homogenous cohort of patients, assessing the impact of FC in the diagnostic algorithm of cPD for the first time. Limitations include a retrospective design; SBCE was evaluated by several readers (inter-observer variability is reported in SBCE); biomarkers were not concomitantly measured with SBCE; and in 20% of patients, FC values were not provided. It should also be acknowledged that the nonsystematic progression to SBCE after the standard workup could have led to selection and referral biases (perhaps due to a more complicated perianal disease or additional clinical descriptives or to physician inclination), thus limiting the generalizability of findings. Nevertheless, the same rate of SBCE positivity was demonstrated in three studies assessing this clinical question so far. A better characterization of cPD patients with positive SBCE may enable improved patient triage to SBCE in the future.

## 5. Conclusions

SBCE was positive in over a quarter of patients with cPD and a negative workup for CD. FC levels correlated with the degree of inflammation defined by the LS. However, low FC was less sensitive to rule out CD. These findings suggest that SBCE should be considered for patients with cPD even after a negative standard workup.

## Figures and Tables

**Table 1:** Biomarkers.

	*n*/N (%)	Range	Median	IQR
Hemoglobin gr/dL	90/91 (99.0)	9.4–17.2	14.3	13.3–14.8
Hemoglobin ≥ 12 gr/dL	84/90 (93.3)			
Albumin gr/dL	85/91 (93.4)	3.3–5.0	4.1	3.9–4.4
Albumin ≥ 3.6 gr/dL	79/85 (92.9)			
CRP mg/dL	84/91 (92.3)	0–17.9	0.43	0.14–1
CRP ≤ 1 mg/dL	64/84 (76.2)			
Fecal calprotectin µg/g	71/91 (78.0)	4–1503	29	10–105
>50 µg/g	29/71 (40.8)			
>100 µg/g	18/71 (25.4)			
>300 µg/g	11/71 (15.5)			

IQR—interquartile range.

**Table 2:** SBCE findings.

Positive SBCE—LS ≥ 135, *n* (%)	24 (26.37%)
Total LS value, median (IQR)	675 (225–1518)
Segmental LS	
SB1 involvement, *n* (%)	11/24 (45.83)
SB1 score value, median (IQR)	112.5 (0–1125)
SB2 involvement, *n* (%)	13/24 (54.16)
SB2 score value, median (IQR)	135 (0–843.8)
SB3 involvement, *n* (%)	22/24 (91.66)
SB3 score value, median (IQR),	506 (225–1518)
Proximal SB inflammation (involved SB1 + SB2), *n* (%)	16/24 (66.66)
Severity of small-bowel inflammation	
Mild inflammation (LS 135–790), *n* (%)	14/24 (58.4)
Mild inflammation, score value, median (IQR),	225 (225–506)
Moderate–severe inflammation (LS ≥ 790), *n* (%)	10/24 (41.6)
Moderate–severe inflammation (LS ≥ 790), median (IQR)	1518 (900–1812)

IQR—inter-quartile range; LS—Lewis Score; SB—small bowel; SB1,2,3—proximal, middle, and distal tertiles according to the capsule transit time along the small bowel; SBCE—small-bowel capsule endoscopy.

**Table 3:** Diagnostic accuracy of FC for the presence of a positive SBCE.

	Sensitivity	Specificity	Positive Predictive Value	Negative Predictive Value	Overall Diagnostic Accuracy
FC = 50 µg/g	55%	64.7%	35.8%	80.1%	62.1%
	(31.2–76.9)	(50.1–77.6)	(24.5–49)	(70.4–87.2)	(49.8–73.4)
FC = 100 µg/g	40.0%	80.4%	42.2%	78.9%	69.7%
	(19.1–63.0)	(66.9–90.2)	(25.2–61.3)	(71.8–84.6)	(57.7–80.1)
FC = 300 µg/g	30%	90.2%	52.3%	78.2%	74.3%
	(11.9–54.3)	(78.6–96.7)	(27.4–76.1)	(72.7–82.9)	(62.6–84.0)

FC—fecal calprotectin; SBCE—small-bowel capsule endoscopy.

## Data Availability

The data underlying this article cannot be shared publicly due to privacy requirements of participating centers. The data will be shared on reasonable request to the corresponding author.
